# High-throughput PCR assay design for targeted resequencing using primerXL

**DOI:** 10.1186/s12859-017-1809-3

**Published:** 2017-09-06

**Authors:** Steve Lefever, Filip Pattyn, Bram De Wilde, Frauke Coppieters, Sarah De Keulenaer, Jan Hellemans, Jo Vandesompele

**Affiliations:** 1 Center for Medical Genetics, Ghent University, De Pintelaan 185, 9000 Ghent, Belgium; 2 pxlence, 9200 Dendermonde, Belgium; 3 Cancer Research Institute Ghent (CRIG), 9000 Ghent, Belgium; 4 Bioinformatics Institute Ghent (BIG), 9000 Ghent, Belgium; 5 Present address: Biogazelle, Technologiepark 3, 9052 Zwijnaarde, Belgium; 6 Present address: NXTGNT, UGent, FFW Building 3th floor, Ottergemsesteenweg 460, 9000 Ghent, Belgium; 7 Present address: Ontoforce, Ottergemsesteenweg-Zuid 808, 9000 Ghent, Belgium

**Keywords:** Primer design, Targeted resequencing, Variant confirmation next generation sequencing, Sanger sequencing, PCR assay

## Abstract

**Background:**

Although the sequencing landscape is rapidly evolving and sequencing costs are continuously decreasing, whole genome sequencing is still too expensive for use on a routine basis. Targeted resequencing of only the regions of interest decreases both costs and the complexity of the downstream data-analysis. Various target enrichment strategies are available, but none of them obtain the degree of coverage uniformity, flexibility and specificity of PCR-based enrichment. On the other hand, the biggest limitation of target enrichment by PCR is the need to design large numbers of partially overlapping assays to cover the target.

**Results:**

To overcome the aforementioned hurdles, we have developed primerXL, a state-of-the-art PCR primer design pipeline for targeted resequencing. It uses an optimized design criteria relaxation cascade and a thorough downstream in silico evaluation process to generate high quality singleplex PCR assays, reducing the need for amplicon normalization, and outperforming other target enrichment strategies and similar primer design tools when considering assay quality, coverage uniformity and target coverage. Results of four different sequencing projects with 2348 amplicons in total covering 470 kb are presented. PrimerXL can be accessed at www.primerxl.org.

**Conclusion:**

PrimerXL is an state-of-the-art, easy to use primer design webtool capable of generating high-quality targeted resequencing assays. The workflow is fully customizable to suit every researchers’ needs, while an innovative relaxation cascade ensures maximal target coverage.

**Electronic supplementary material:**

The online version of this article doi:(10.1186/s12859-017-1809-3) contains supplementary material, which is available to authorized users.

## Background

Massively parallel sequencing has opened the path towards personalized genomics but the current sequencing cost and limitations in data-analysis impede the wide use of whole-genome sequencing in a clinical context. However, by focusing the power of this new technology on a region of interest through specific target enrichment and pooling multiple samples in one sequencing run, the sequencing cost can be reduced dramatically [1, 2]. Different target enrichment strategies have emerged, each with their own benefits and limitations. Enrichment through hybridization, either array- or solution-based [2–5], is capable of capturing larger target regions but lacks the flexibility of PCR-based approaches in the context of specificity (when pseudogenes are known for the gene of interest), regional GC content and gene panel contents. The latter is mainly the case for array-based hybridization enrichment, since addition of targets to an existing panel requires the redesign of the array.. In addition, on-array target enrichment requires specialized instruments and relatively large amounts of DNA. Although enrichment by PCR seems to outperform hybridization enrichment strategies when looking at specificity and coverage uniformity, this strategy is less frequently used thus far due to the fact that performing many PCRs in parallel may not be straightforward and that multiple assays need to be designed to cover the complete region of interest [2, 5]. More recent high-throughput PCR strategies, such as micro-droplet PCR by Raindance [6], nanoliter SmartChip reactions by WaferGen Biosystems and Access arrays by Fluidigm, could bring PCR enrichment into the mainstream. The large number of parallel small-volume reactions in these new platforms substantially reduces the turnaround time, cost and the required amount of input DNA. However, a bottleneck remains, i.e. the design of a large number of PCR assays. While various primer design software packages are available, most are not suited for amplicon generation in the context of targeted resequencing of entire genes. ExonPrimer [7] expects the input of a cDNA and matching genomic sequence to extract intron-exon boundaries. Customization is limited and some essential downstream primer pair evaluations, such as specificity and secondary structure assessment, are lacking. The Optimus Primer pipeline [8] is more user friendly, accepts both official gene symbols and chromosomal regions as input and is able to perform up to four design criteria relaxation steps to maximize target coverage. The presence of secondary structures in primer annealing sites is not evaluated and the process of splitting up larger exons or regions in smaller fragments to be processed in parallel, limits both the possibility to optimize target coverage (i.e. minimizing amplicon overlap and near-target coverage) and to reduce the number of amplicons. To address these shortcomings, we have developed primerXL, a state-of-the-art high-throughput primer design pipeline for massively parallel targeted resequencing. It employs an optimized design parameter relaxation cascade and multiple primer pair quality control analyses, generating high quality and robust singleplex PCR assays resulting in uniform sequencing coverage. In addition, an accompanying straightforward and easy to use web tool has been developed for users to fully customize their designs, starting with a gene symbol, transcript ID or chromosomal region as input. PrimerXL is available at [9].

## Implementation

### PrimerXL workflow

PrimerXL is a fully automated, modular pipeline, making it easy to add, remove or change features. Its heart consists of a copy of the Ensembl core and variation databases [10] for a number of selected species and custom MySQL tables for storage of design requests and results. The power of primerXL lies in the fact that it combines the proven primer3 primer design engine [11] with optimized parameter relaxation and a thorough downstream in silico assay evaluation. PrimerXL generates high quality primer pairs, omitting the need for laborious wet-lab assay testing and optimization while covering close to 100% of the target region.

**Fig. 1:**
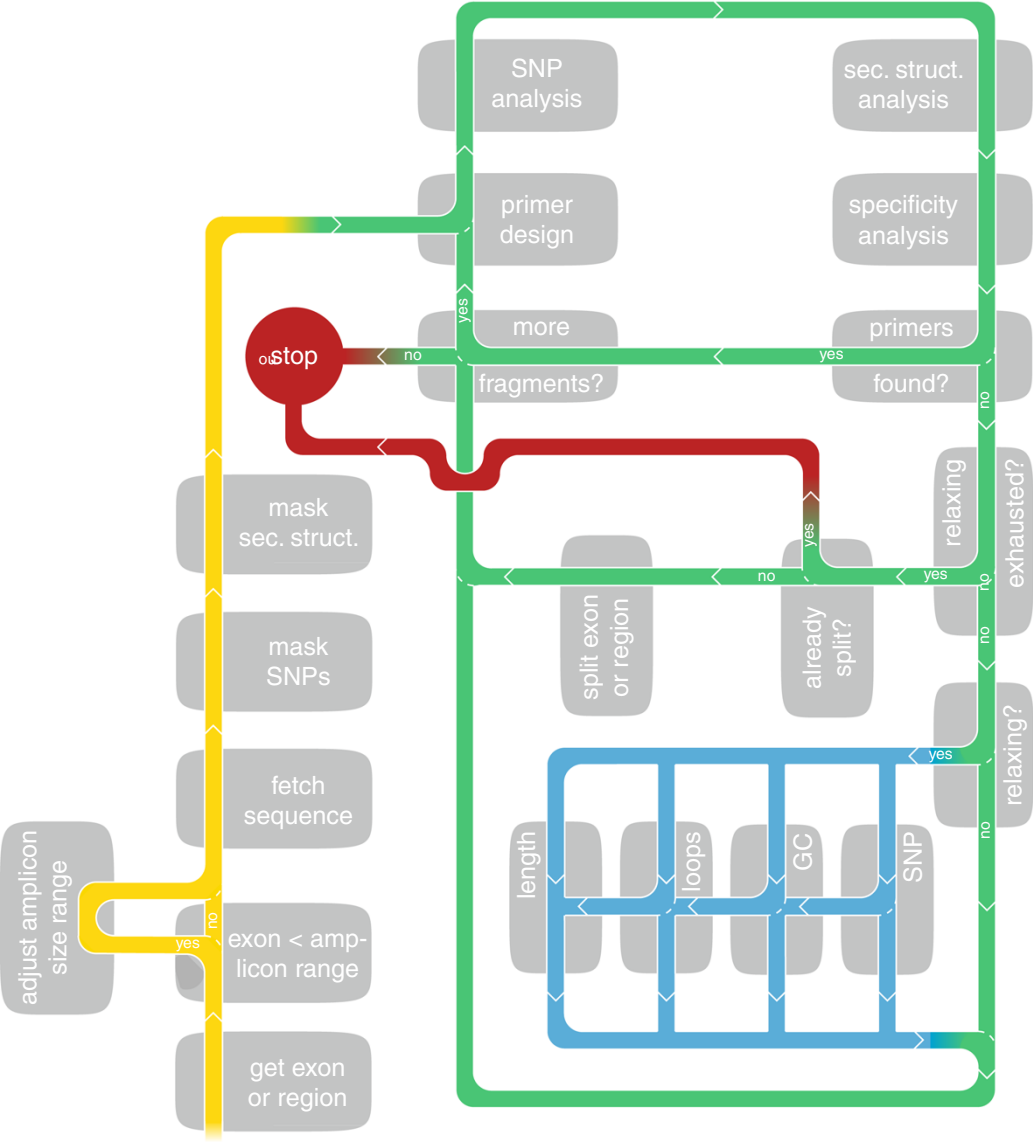
PrimerXL targeted resequencing PCR design pipeline. Schematic representation of the primerXL pipeline functionality. Tasks performed by the parent process are marked in yellow, tasks executed by the child processes in green and blue. The design criteria relaxation cascade is shown in blue. Following retrieval of the sequence of interest (and adjustment of the amplicon size range for small regions), SNPs and secondary structures are masked. Next Primer3-based primer design is initiated and a SNP- and secondary structure analysis is performed on each resulting assay to remove assays harboring these features in their primer annealing sites. If a successful primer pair remains after specificity assessment, the next target region is processed or the design is terminated. On the other hand, if no successful assays remain, design parameters are relaxed (specificity-, SNP- and secondary structure analysis stringency, GC content, amplicon length, …) and primer design is attempted once again. If all parameters have already been relaxed to their fullest extent without the generation of a successful primer pair, the design process is terminated

The workflow of the primerXL pipeline is simple and completely customizable. Once a design request has been submitted by way of a chromosomal region, gene symbol or transcript ID, primerXL starts by retrieving all relevant DNA sequences. In case a gene symbol is supplied, the input sequence will consist of the unique coding sequences of all corresponding transcripts. Next, features such as single nucleotide polymorphisms (SNP) and secondary structures, known to have a negative effect on amplification efficiency [12–14] are masked in the DNA sequences based on data of the Ensembl variation database [10] and results of a UNAfold analysis [15], respectively. Excluding SNPs and secondary structures from primer annealing sites is essential since these can hamper proper hybridization of a primer to its target sequence. This may result in allelic drop out and reduced amplification. In order to increase the throughput of the pipeline, parallel child processes are created for each exon or chromosomal region to perform the primer design and the downstream in silico evaluation for the corresponding target region. Tasks executed by the child process are indicated in blue and green in Fig. 1.

**Fig. 2:**
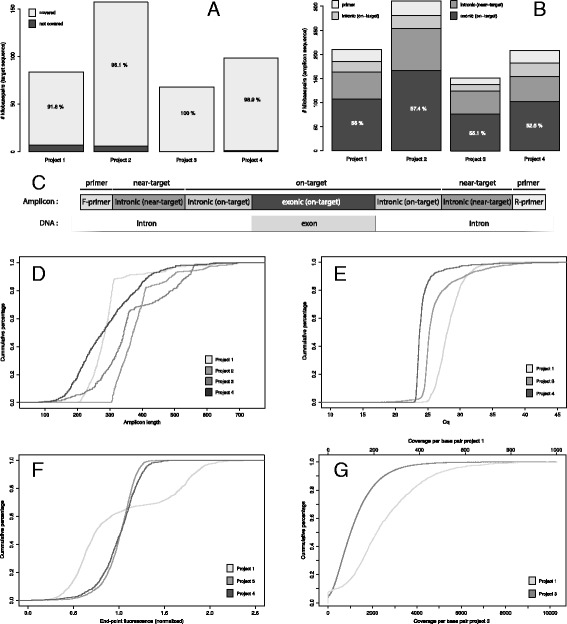
Amplicon and sequencing statistics. Graphical representation of the amplicon and sequencing statistics of four sequencing projects. **a** Target coverage efficiency, **b** Distribution of the amplicon sequence, **c** Schematic displaying the different on- and off-target portions of an amplicon, **d** Cumulative distribution of the amplicon length, **e** Cumulative distribution of the Cq values (amplicons of Project 2 were tested by regular PCR), **f** Cumulative distribution of the end-point fluorescence and **g** Cumulative distribution of the sequencing coverage

In order to cover putative mutations in donor and acceptor splice sites and to accommodate lower sequencing quality at the start of a read – especially in the case of Sanger sequencing – primerXL offers the possibility to include 5′ and 3′ intronic regions in the target to be sequenced. As a consequence, when describing our results, amplicon sequences will be divided in three parts. The exonic on-target region is the part of an amplicon covering the actual region of interest, being the exon or the chromosomal location of interest. The extra-included 3′ and 5′ intronic regions are referred to as intronic on-target, while the rest of the amplicon is defined as intronic near-target (Fig. 2g). Maximizing target coverage while minimizing the number of required amplicons is achieved by means of an optimized relaxation cascade (indicated in blue in Fig. 1). First, using the parameters set by the user, primerXL will try to span the entire fragment under consideration using a single amplicon. If this fails (for example because the size of the fragment is exceeding the maximum set amplicon length), design settings will be relaxed until a targeted resequencing assay is successfully generated or all relaxation options have been exhausted. The default relaxation cascade comprises – in order of their execution – allowing SNPs in primer annealing sites except for a predefined region on the primer 3′ end [12, 13], increasing the GC clamp size (i.e. the number of G/C nucleotides in the last 5 base pairs on the 3′ end of a primer) incrementally to its maximum [16], tolerating small secondary structures in annealing sites, stepwise (100 bp per step) increasing amplicon length to a maximum of 1.5 times the original upper length limit, enlarging the allowed annealing temperature range (stepwise by 0.5 °C with a maximum range of 5 °C) and finally relaxing specificity analysis by reducing the maximum number of mismatches allowed in potential annealing sites. When all possible relaxation options and their combinations have been considered without the successful generation of an assay, primerXL will redefine its target. This process depends on the size of the fragment. If the size of the fragment is larger than the minimum set amplicon length, primerXL will define a region on the 3′ end of the fragment as the new target, reset the relaxation cascade and attempt to find an assay amplifying only this piece of the fragment. However, if the fragment is already smaller than the minimum amplicon length, primerXL will shift its focus to the 5′ end of the fragment (i.e. the part of the fragment that remained after target-redefinition will now become the fragment of interest) and restart the whole procedure. The design process ends when the initial fragment has been covered completely or when the size of the 5′ end of the fragment from the previous design step is smaller than the minimum amplicon length and no assays can be found to cover it. In addition to fragmenting large regions in this way, primerXL can also dynamically adjust the amplicon size range for smaller exons, thus maximizing the target size that can be sequenced in one run by limiting the size of undesired near-target sequences.

Three types of downstream in silico assay evaluation procedures are incorporated in primerXL. The first type assesses the presence of SNPs in primer annealing sites. In the most stringent setting, assays harboring SNPs in primer annealing sites are filtered out by masking them by means of Primer3 sequence quality scores. If SNP relaxation is enabled, this feature ignores the quality scores during Primer3 design, but allows the use of the scores after design to annotate SNP presence for each assay individually. This can be used to guarantee that no SNPs are present in a predefined number of bases at the 3′ end of a primer. This number can be set by the user but we observed that mismatches are well tolerated, except in the last five base pairs on the 3′ end of a primer [17]. The location of secondary structures in primer annealing sites is assessed in the same way as for SNPs, by taking into account sequence quality scores in Primer3. However, since secondary structures can vary between the final amplicon and the original template in its sequence context, we cannot rely on these quality scores solely. Both in the most stringent and relaxed mode, it is important to reanalyze the secondary structure content of the amplicon by way of a second type of post-design evaluation to ensure that primer annealing sites are free of secondary structures. The final downstream in silico assay evaluation uses a local Bowtie implementation to determine the specificity of the primer pair by aligning the forward and reverse primer sequences against the reference genome, in combination with a user-defined maximum allowed number of mismatches [18]. By using a higher mismatch number, potential non-specific annealing sites harboring mismatches will be returned along with the perfect matching regions. This allows to discard assays with a non-specific amplification potential by accepting primer pairs that only return the perfect hits, even when using higher mismatch numbers to screen for potential non-specific amplification. Together, these three detailed downstream in silico assay evaluations result in high quality singleplex primer pairs. Due to the modular nature of primerXL, additional evaluations can be added.

### User interface

To allow users to easily customize and submit primer design jobs, a user-friendly web-interface was created. PrimerXL is available for *Homo sapiens, Mus musculus, Rattus norvegicus, Oryza sativa, Arabidopsis thaliana, Equus caballus, Zea mays, Triticum aestivum, Bos Taurus, Sus scrofa* and *Danio rerio.* Additional organisms can be added upon request. Following specification of the project name and species selection, the user will be requested to choose the template for which tiling assays should be designed. The required identifier (gene, transcript or chromosomal location) depends on the chosen template. For a cDNA template, a gene or transcript identifier will be asked; for a genomic DNA (gDNA) template, an option to enter a chromosomal location is available. If a supplied identifier results in multiple hits when querying the database, matching suggestions will be shown to the user (gene and/or transcript identifiers depending on the template) in order to select the preferred one. All user adjustable primerXL settings, including the parameters of the third-party software packages such as primer3, UNAFold and Bowtie, are available online. Optimized – but customizable – default settings have been predefined for Sanger sequencing, Roche 454 and Illumina resequencing. After submission, the design request is stored in a database and executed on the primerXL server. Currently, no locally installable version of the tool is available. Once primer design has been completed, results are emailed to the user and can be viewed online. Non-commercial access to primerXL is available after registration and the number of targeted resequencing requests has been limited to one per user per month. PrimerXL is available at [9].

## Results

In our department, primerXL has been extensively used for targeted re-sequencing. One of the first projects entailed the Roche 454 sequencing of 15 genes involved in hereditary deafness (Project 1 using primerXL v0.8 (see Table 1) – 83 Kb) and generated 625 amplicons targeting 91.76% of the coding sequences of all corresponding transcript variants [19]. When running the same genes using the latest version of primerXL (v1.0), target coverage increased to 95.7%. Sixty five percent of the amplicon sequence, 185 Kb in total, was on target with over 90% of the amplicons with a length between 200 and 350 bp, well suited – at that time – for Roche 454-FLX sequencing. Upon assay evaluation using qPCR (mean Cq value: 28.9 ± 2.76), 17 assays failed (2.7%) due to no or limited amplification (Cq > 35) while another 40 amplicons were finally not included in the sequencing for being too long (> 500 bp). End-point fluorescence uniformity was high (93.3% within 2-fold difference of the mean), meaning that no concentration normalization is needed for these amplicons as they will result in a uniform coverage [20]. This was confirmed upon sequencing on a Roche 454-FLX instrument (3 samples on a conventional run) with 70% of the targeted bases within 2-fold above and 2-fold under the mean coverage of 240 reads/base (89.2% within 5-fold above and 5-fold under the mean coverage). None of the amplicon associated parameters – such as GC content, overall Gibbs free energy and length – were correlated with assay performance or sequencing coverage (Additional file 1: Figure S1A-C). However, primer pair specificity and secondary structures in primer annealing sites were shown to have an effect on sequencing coverage. Assays harboring secondary structures in at least on of its primer annealing sites tend to result in lower sequencing coverage (Additional file 1: Figure S1D). Similarly, sequencing coverage is significantly influenced by the assay specificity level, calculated as described in previous work [21]. This is to be expected as lower specificity means that the non-specific products take away a fraction of the sequencing capacity, leaving fewer reads for the amplicon of interest (Additional file 1: Figure S1E). A setup, similar to the one applied for project 1, was used for targeting 24 genes (Project 2 using primerXL v1.0–157 Kb) used in a diagnostic setting where sequencing is planned by Roche 454 technology. The primer design resulted in 723 amplicons covering 96.1% of the target with high amplicon length uniformity (84.5% between 300 and 450 bp). Currently, 638 amplicons have been tested by PCR with a (strict definition of) success rate of 87% (79 non-specific assays and 6 assays with no amplification). Comparison between these two projects (Project 1 and 2) clearly shows the added value of the relaxation. Whereas 91.76% target coverage was achieved by manually relaxing the design settings, implementation of automatic relaxation increased this percentage to 96.1%. Another approach was used in a third project sequencing 23 Leber congenital amaurosis (LCA) and Retinitis Pigmentosa (RP) genes (Project 3 using primerXL v0.9–132 Kb) on the Illumina GAIIx system [22]. Since dynamic optimization was not implemented yet in version 0.9, two primer design rounds with different amplicon sizes (400 bp and 600 bp) were performed from which amplicons were selected to optimally cover all exonic sequences of the corresponding RefSeq transcripts. Both design approaches gave a 96% target coverage, the 600 bp designs showed a higher intronic near-target percentage as expected (35% versus 24% for the 400 bp designs). In total, 375 amplicons covering the RefSeq transcripts of 16 LCA genes (67 Kb) were ligated and fragmented prior to sequencing. Of these amplicons, 96.5% had a Cq below 35 (mean Cq value: 26.4 ± 3.01) while 99.2% had an end-point fluorescence within 2-fold difference of the mean. Coverage analysis of 12 samples sequenced on an Illumina GAIIx instrument (1 lane) indicated that 59% of the targeted bases are within 2-fold above and 2-fold under the mean coverage of the mean coverage of 1232 reads/base (88.6% within 5-fold above and 5-fold under the mean coverage). With respect to coverage analysis, similar conclusions as to the ones for project 1 can be drawn. Assay specificity and secondary structures in primer annealing sites seem to have an effect on sequencing coverage, while no impact can be observed for amplicon GC content, overall Gibbs free energy and amplicon length (Additional file 2: Figure S2A-E). The best results were obtained in the fourth project sequencing 558 exons (Project 4 using primerXL v1.0–98 Kb). Nearly 98.9% of the targets were covered using 625 amplicons of which only 8 (1.2%) resulted in a Cq higher than 35 (mean Cq value: 24.3 ± 1.88). Although no sequencing data are available yet, 96.8% of the assays showed an end-point fluorescence within 1.5 times the mean. The results from these four projects are summarized in Table 2 and Fig. 2.

**Table 1:** PrimerXL feature overview in function of pipeline version

Version	Relaxation cascade	Dynamic target adjustment
0.8		
0.9	V	
1.0	V	V

Differences in primerXL features between the various versions used in this study. The term “dynamic target adjustment” refers to the ability of primerXL to automatically adjust the amplicon size range for smaller exons, thus reducing the amount of intronic near-target sequence

**Table 2:** Projects statistics overview

		Amplicon design							PCR			Sequencing		
	Target size	# amplicons	Total amplicon sequence	Exonic (on-target)	Intronic (on-target)	Intronic (near-target)	F-primer	R primer	Assays tested	Failed (No amplification)	Multiple bands	Assays sequenced	No reads	% bp within 2 × mean coverage
Project 1	83 Kb	625	185 Kb	108 Kb (58.1%)	22 Kb (11.7%)	56 Kb (30.2%)	13 Kb	13 Kb	626	17 (2.7%)	NA	569	8 (1.4%)	70%
Project 2	157 Kb	723	281 Kb	167 Kb (59.3%)	27 Kb (9.7%)	87 Kb (31.0%)	15 Kb	15 Kb	638	6 (0.9%)	79 (12.4%)	NA	NA	NA
Project 3	67 Kb	375	137 Kb	77 Kb (56.2%)	14 Kb (9.9%)	47 Kb (33.9%)	8 Kb	8 Kb	375	0 (0.0%)	NA	375	18 (5%)	59%
Project 4	98 Kb	625	182 Kb	102 Kb (56.1%)	29 Kb (15.7%)	51 Kb (28.2%)	13 Kb	13 Kb	625	8 (1.3%)	NA	NA	NA	NA

Details of the different projects showing the design target coverage, success rate when testing the assays on (q)PCR and sequence coverage

All aforementioned experiments were performed with high-quality DNA, allowing longer amplicon lengths. For formalin-fixed paraffin embedded (FFPE) tissue samples resulting in fragmented DNA, assays generating shorter amplicons are commonly applied. While the degree of fragmentation is dependent on the age of the sample and the type and duration of fixation, in general assays shorter than 300 nucleotides are recommended. To determine how primerXL copes with shorter amplicon size ranges, we designed assays for 31 genes – i.e. the 15 deafness genes from project 1 and 16 LCA genes included in project 3 – using 80–200, 200–300 and 80–300 basepair size ranges. Results depicted in Additional file 3: Figure S3, show that the coverage for these size ranges is significantly lower than what could be obtained with longer products in the aforementioned projects. Seeing that the failure rate for longer exons (larger than 1 kb) of 27.5% (11/40 failed exons) is larger than the failure rate for shorter exons (3.56% or 24/674 exons), this could partly be explained by the hard-coded three-day design limit embedded in primerXL combined with the more limited design space inherent with shorter amplicon sizes. Indeed, the more stringent design space further increases the already longer design time associated with larger exons, pushing them toward the design wall-time resulting in an increased number of them to end prematurely without successful assays. To circumvent this, users can split up larger exons manually although this will most likely result in suboptimal tiling. This could be confirmed by splitting up the exons larger than 1 kb that failed to generate assays using the 80–300 basepair setup, into fragments of approximately 500 bp. Using this approach, the pipeline was able to cover 67.28% of these fragments bringing the total coverage for the 31 genes up to 90.12%. Shorter amplicon assay design is likely also to negatively impact the specificity of the design (given the higher design constraint and smaller design space) and as such make designs for genes with pseudogenes somewhat more challenging.

**Fig. 3:**
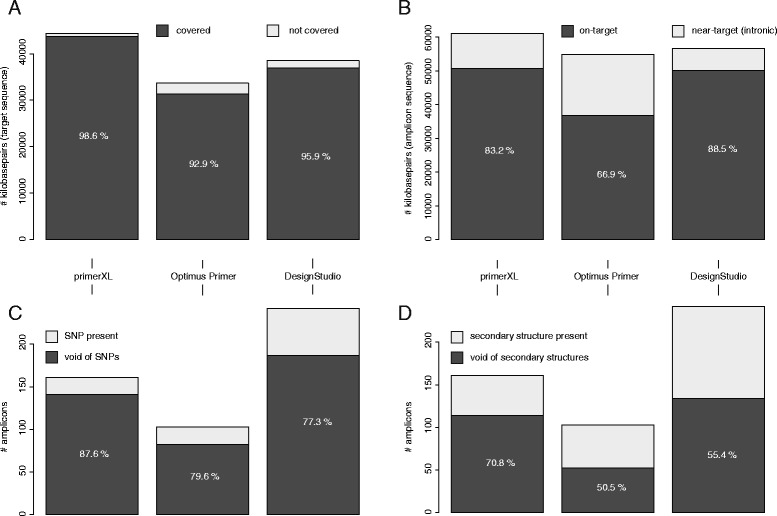
Targeted resequencing assay statistics. Graphical representation of the targeted resequencing assay statistics for five genes (*SACS*, *SETX*, *APTX*, *ANO10*, *CYP27A1*) designed using three primer design tools (primerXL, Optimus Primer and Illumina DesignStudio): **a** Target coverage efficiency, **b** Distribution of the amplicon sequence and percentage of amplicons harbouring SNPs (**c**) or secondary structures **d** in primer annealing sites

To assess primerXL performance in comparison to other primer design tools, targeted resequencing assays were designed for five randomly selected genes (*SACS*, *SETX, APTX, ANO10* and *CYP27A1)* using three different tools (primerXL, Illumina DesignStudio and Optimus Primer). Although differences in target region can be observed among the tools – DesignStudio uses the Illumina UCSC iGenomes (hg19), primerXL targets the exons of all known transcript variants of the gene of interest, and Optimus Primer is based on NCBI reference transcripts (RefSeqs) – comparisons can be made by taking into account the corresponding target size when calculating the different parameter percentage values. Here we looked at the percentage of the target each tool was able to cover using targeted resequencing assays, as well as the percentage of the total amplicon sequence that was either on- or near-target. In addition, in silico assay evaluations were performed to assess how each primer design tool copes with features known to affect PCR assay performance such as the presence of SNPs and secondary structures in primer annealing sites [14]. Design settings between the three tools were kept identical where possible. The amplicon size range for both primerXL and Optimus Primer was set at 350 to 450 bp. In contrast to the automatic relaxation cascade in primerXL, relaxed parameters had to be set manually in Optimus Primer (1st relaxation: allow SNPs in primer annealing sites; 2nd relaxation: mask SNPs and increase amplicon size range to 450–550 bp; 3rd relaxation: don’t mask SNPs and increase amplicon size range to 450–550 bp). The Illumina DesignStudio does not allow customization of the amplicon length, nor the adjustment of the relaxation cascade. When looking at the targeted resequencing assays designed by the three primer design pipelines, it is clear that primerXL is able to cover more of its target (98.6% for primerXL versus 92.9% for Optimus Primer and 95.9% for DesignStudio – Fig. 3a), while maintaining a high on-target percentage (83.2%) (Fig. 3b). Although DesignStudio shows the highest on-target ratio (88.5%), the percentage of amplicons harboring SNPs in at least one primer annealing site is more than twice as high compared to primerXL, indicating that the primerXL assays are likely to be more robust and insensitive to natural sequence variation (Fig. 3c). The low on-target percentage for Optimus Primer can be attributed to the fact that all assays were designed using the third relaxation setting, having an increased amplicon size range. Since DesignStudio does not allow retrieval of exact primer locations, 20 bp regions on both the 3′ and 5′ end were considered as primer annealing sites for assays designed by this tool. Figure 3d shows that primerXL also outperforms both other primer design tools with respect to lower frequency of secondary structures in primer annealing regions.

## Conclusion

Although PCR is a cost-efficient, easy and efficient target enrichment strategy for both next generation sequencing as well as large scale Sanger confirmation sequencing, its use is hampered by the lack of tools capable in designing the large number of assays required to cover the target of interest. Our newly developed primerXL pipeline is an easy to use primer design tool for singleplex PCR based targeted resequencing and has proven its value in several projects. A one-pass primerXL design intended for high-quality DNA typically results in amplicons covering at least 96% of the target region with very high coverage uniformity (~ 70% within 2-fold above and 2-fold under the mean coverage), outperforming other hybridization or solution based target enrichment strategies [2]. Also, compared to other primer design tools capable of generating assays for targeted resequencing, primerXL scores better when looking at target coverage (covering all splice variants and with larger fraction on-target), percentage on-target sequence and quality of the designed assays. PrimerXL can be accessed at [9].

## Availability and requirements

**Project name:** primerXL

**Project home page:** http://www.primerXL.org

**Operating system(s):** Platform independent.

**Programming language(s):** Perl, PHP, HTML.

**License: **GNU GPL.

**Any restrictions to use by non-academics:** licence needed.

## Additional files

Additional file 1: Figure S1. Impact of assay features on sequencing coverage in project 1. Scatter plots of the assay sequencing coverage in function of A) the Gibbs free energy, B) the amplicon length and C) the amplicon GC content. Cumulative percentage plots of the assay sequencing coverage in function of D) the secondary structure content in primer annealing sites and E) the assay specificity level. Pearson correlation values and *p* values were calculated using the R functions cor() and ks.test() (Kolmogorov-Smirnov test) respectively. (PDF 795 kb)

Additional file 2: Figure S2. Impact of assay features on sequencing coverage in project 3. Scatter plots of the assay sequencing coverage in function of A) the Gibbs free energy, B) the amplicon length and C) the amplicon GC content. Cumulative percentage plots of the assay sequencing coverage in function of D) the secondary structure content in primer annealing sites and E) the assay specificity level. Pearson correlation values and *p* values were calculated using the R functions cor() and ks.test() (Kolmogorov-Smirnov test) respectively. (PDF 1450 kb)

Additional file 3: Figure S3. Design performance when using amplicon sizes optimized for FFPE samples. Barplots showing target coverage percentages for 31 genes – totaling 242,939 nucleotides – using 80–200, 200–300 and 80–300 basepair design size ranges. (PDF 330 kb)

## Abbreviations

LCA: leber congenital amaurosis
SNP: single nucleotide polymorphism
