# Supplementary figures and images for: High-throughput PCR assay design for targeted resequencing using primerXL
Source: BMC Bioinformatics. 2017 Sep 6;18:400. doi: 10.1186/s12859-017-1809-3 (PMC5588703; doi:10.1186/s12859-017-1809-3)

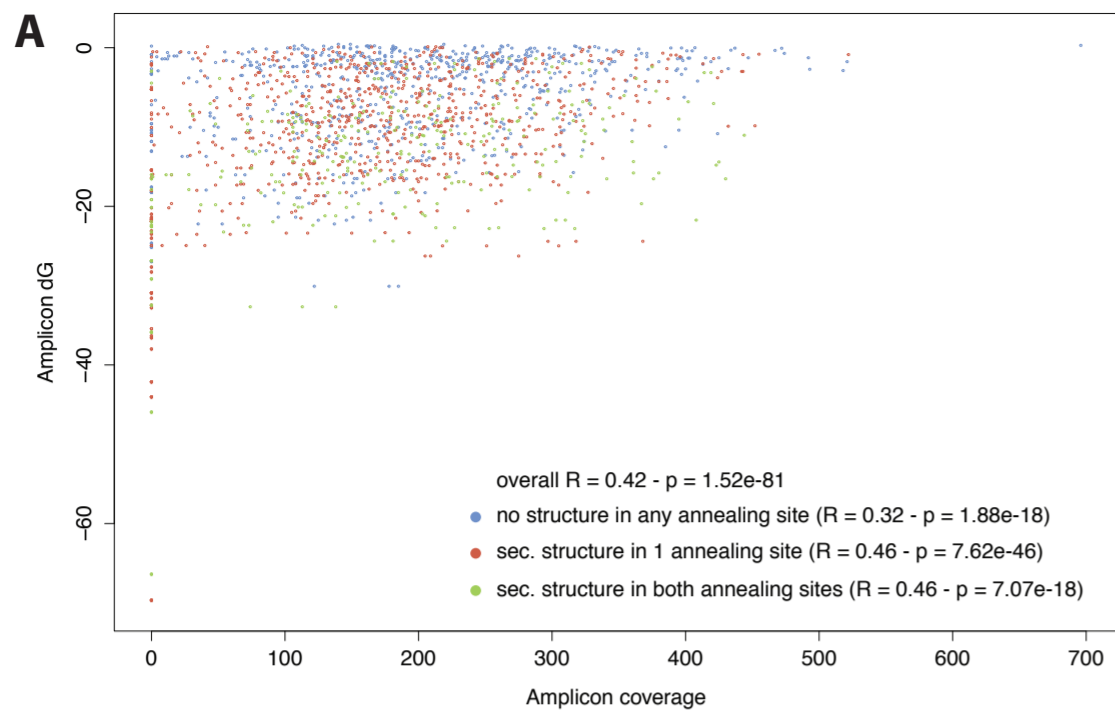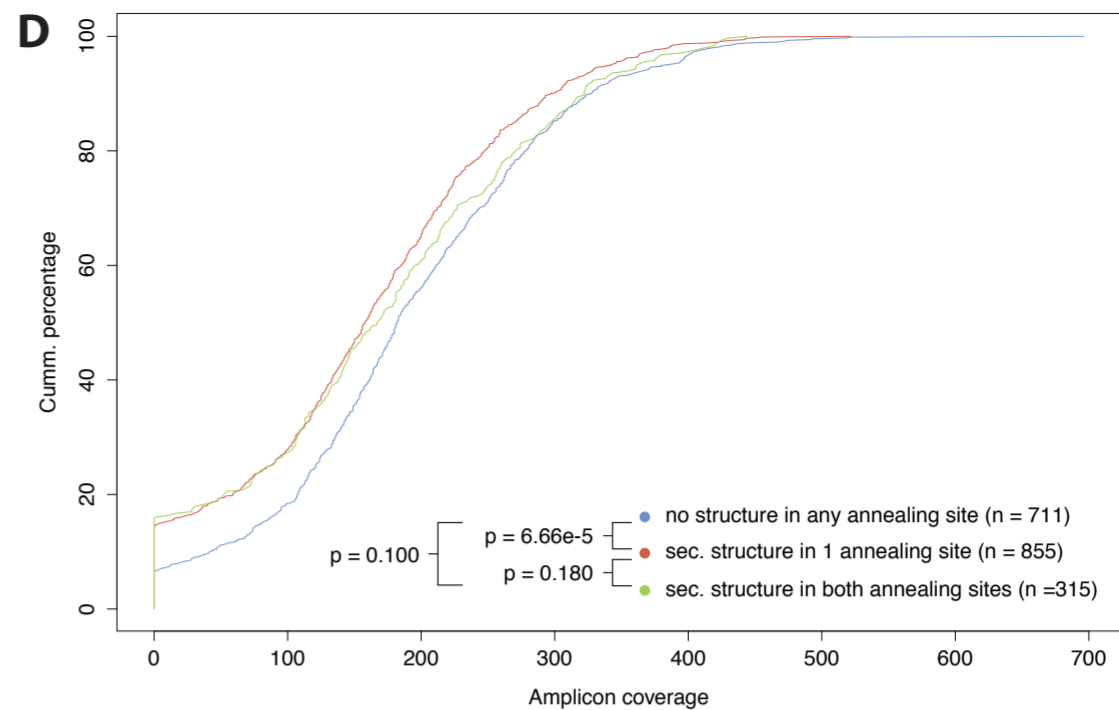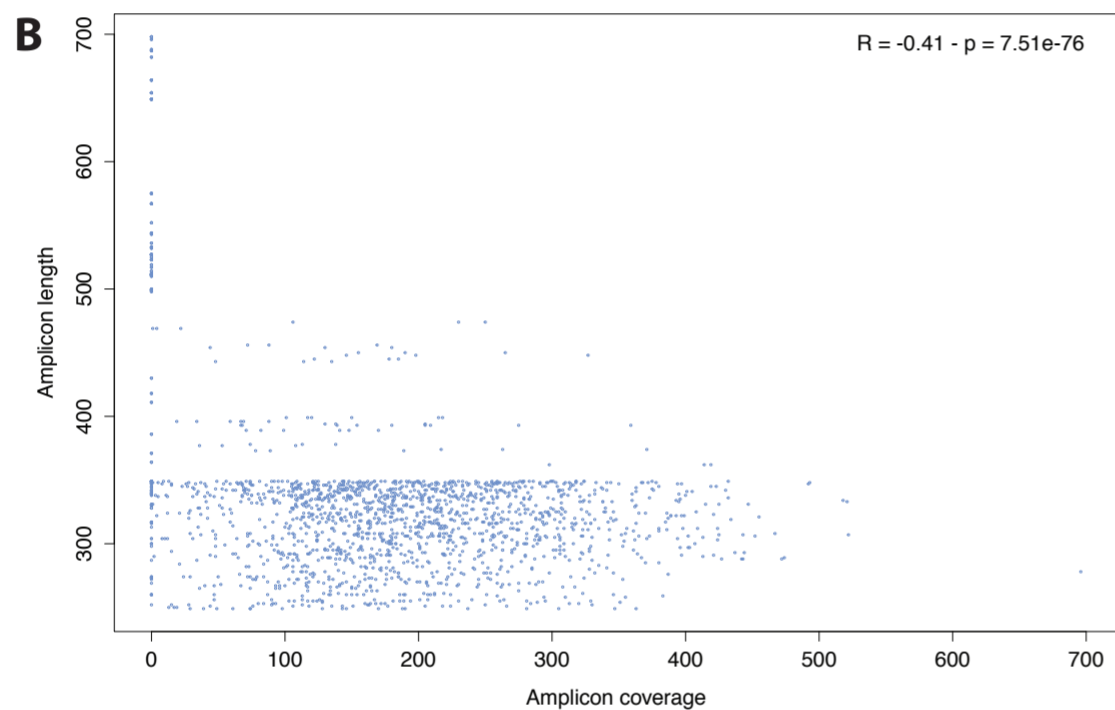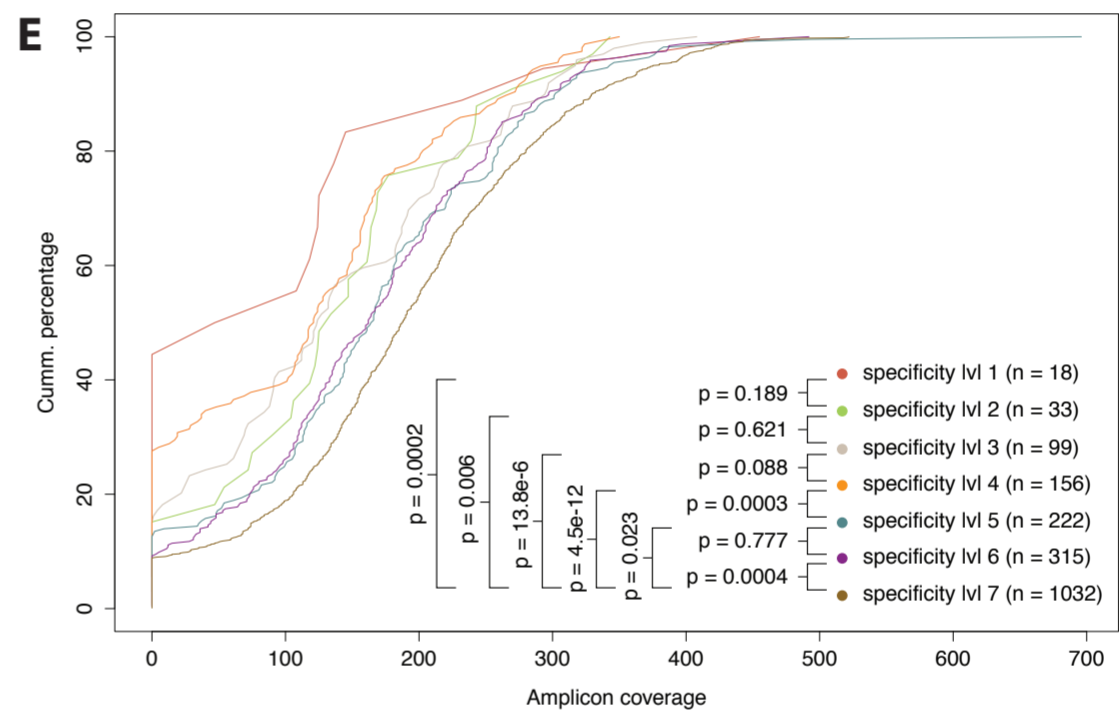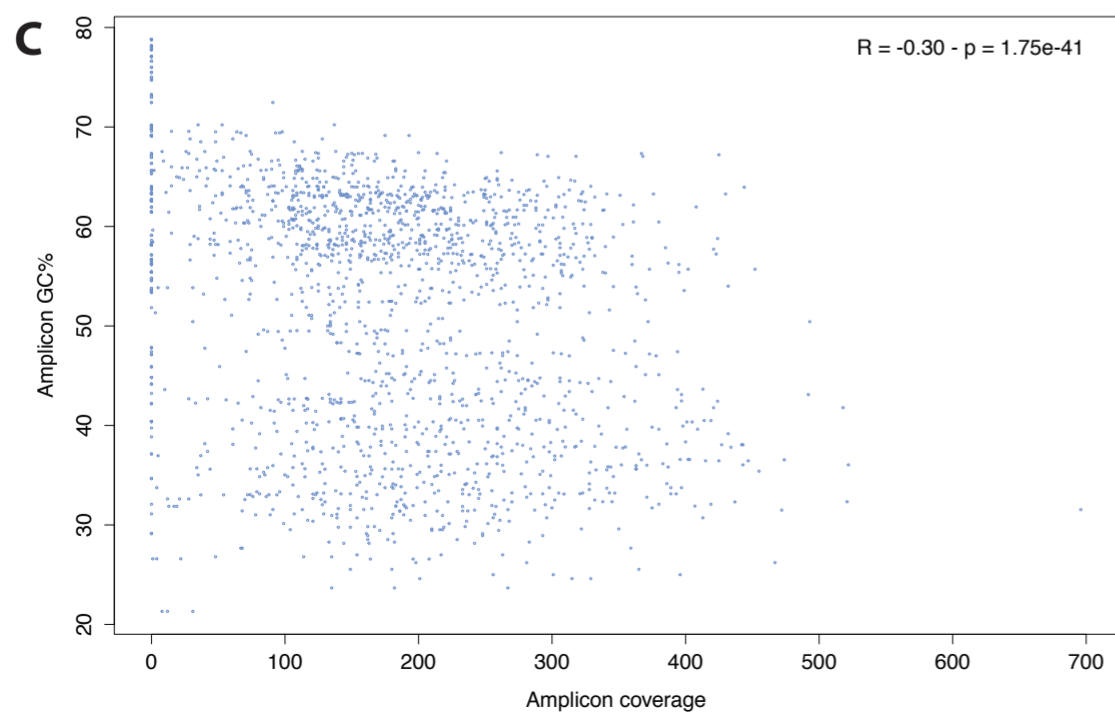

Supplement: Supplementary file 1 — Impact of assay features on sequencing coverage in project 1. Scatter plots of the assay sequencing coverage in function of A) the Gibbs free energy, B) the amplicon length and C) the amplicon GC content. Cumulative percentage plots of the assay sequencing coverage in function of D) the secondary structure content in primer annealing sites and E) the assay specificity level. Pearson correlation values and p values were calculated using the R functions cor() and ks.test() (Kolmogorov-Smirnov test) respectively. (PDF 795 kb) [file 12859_2017_1809_MOESM1_ESM.pdf]

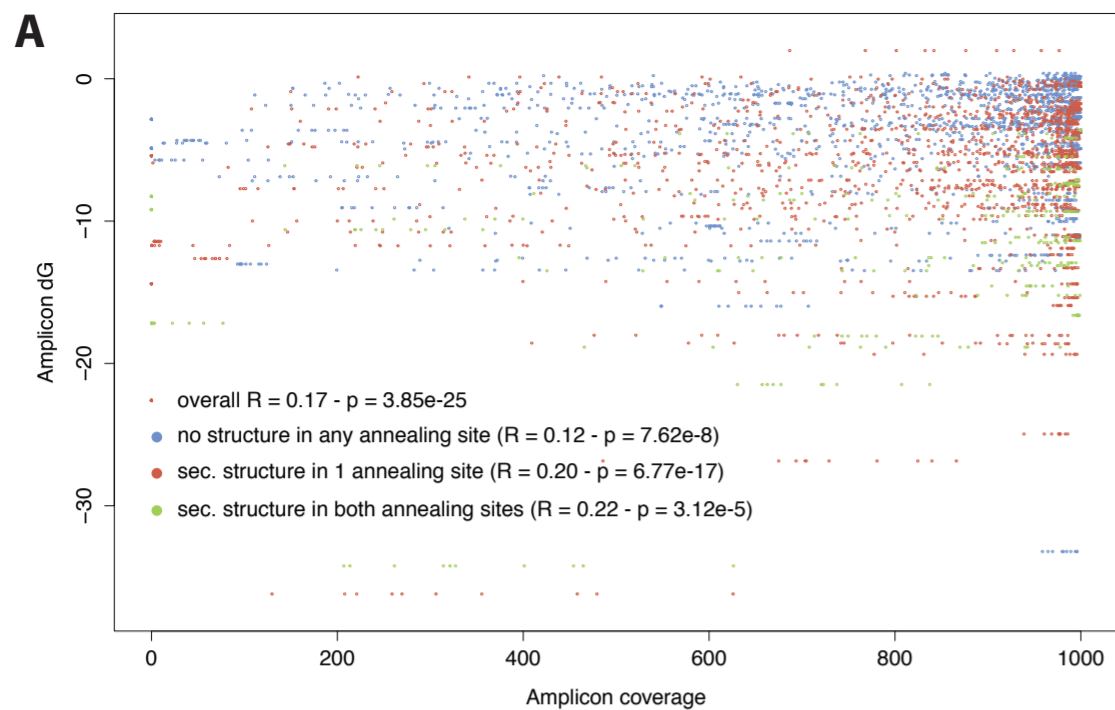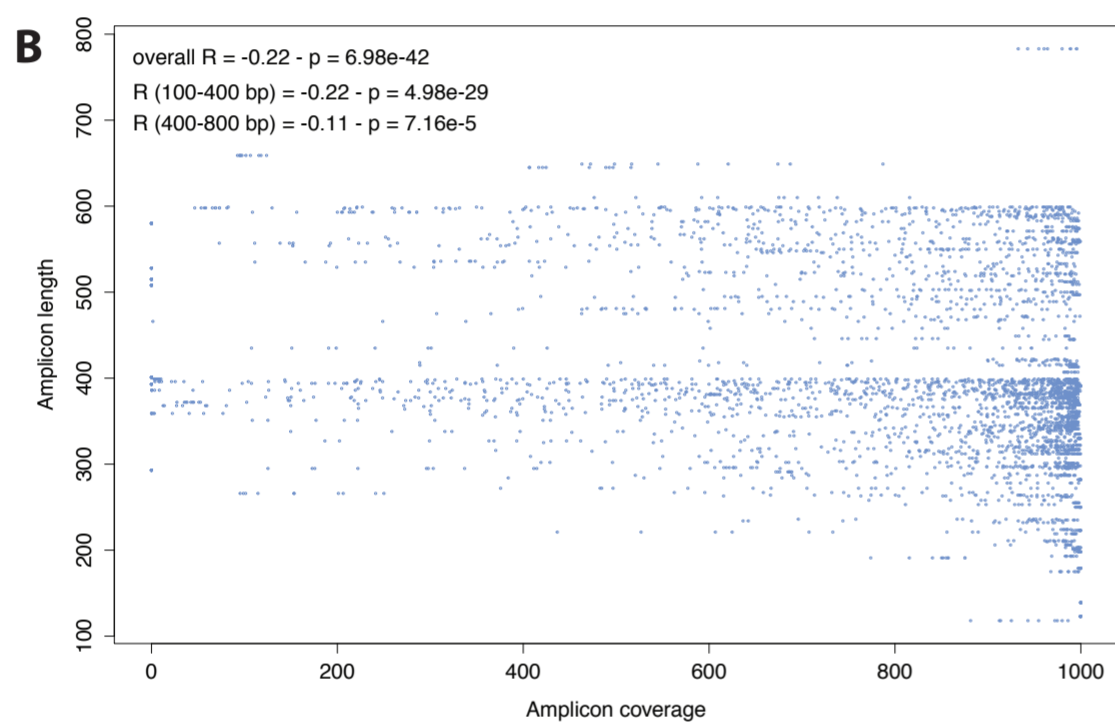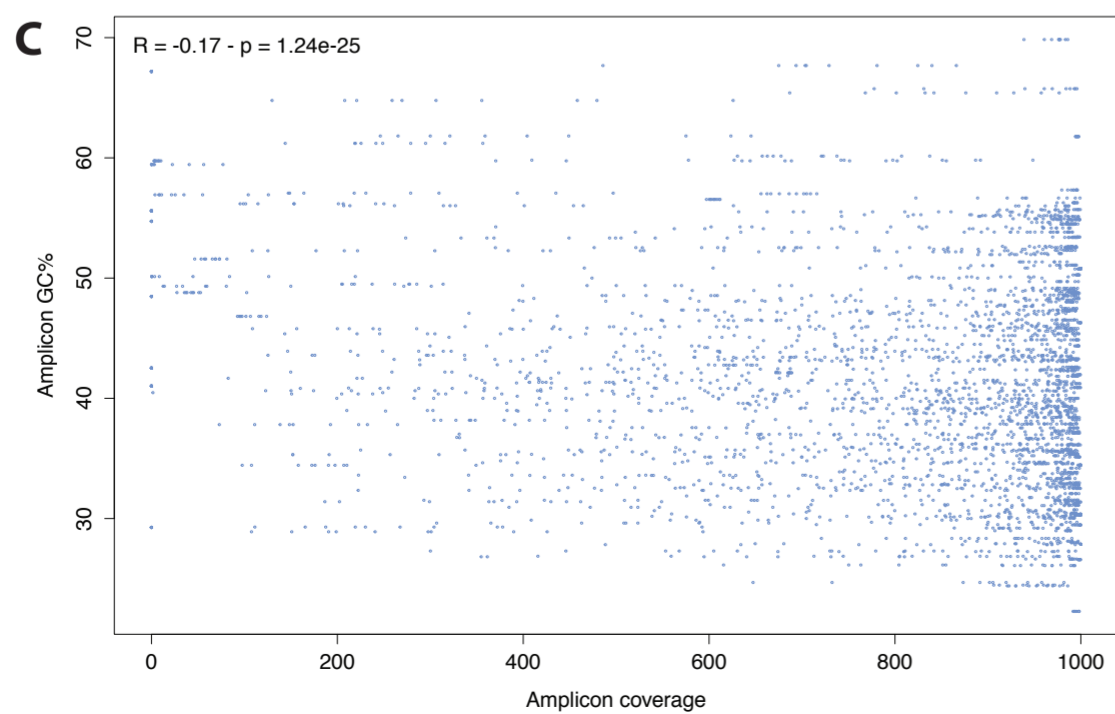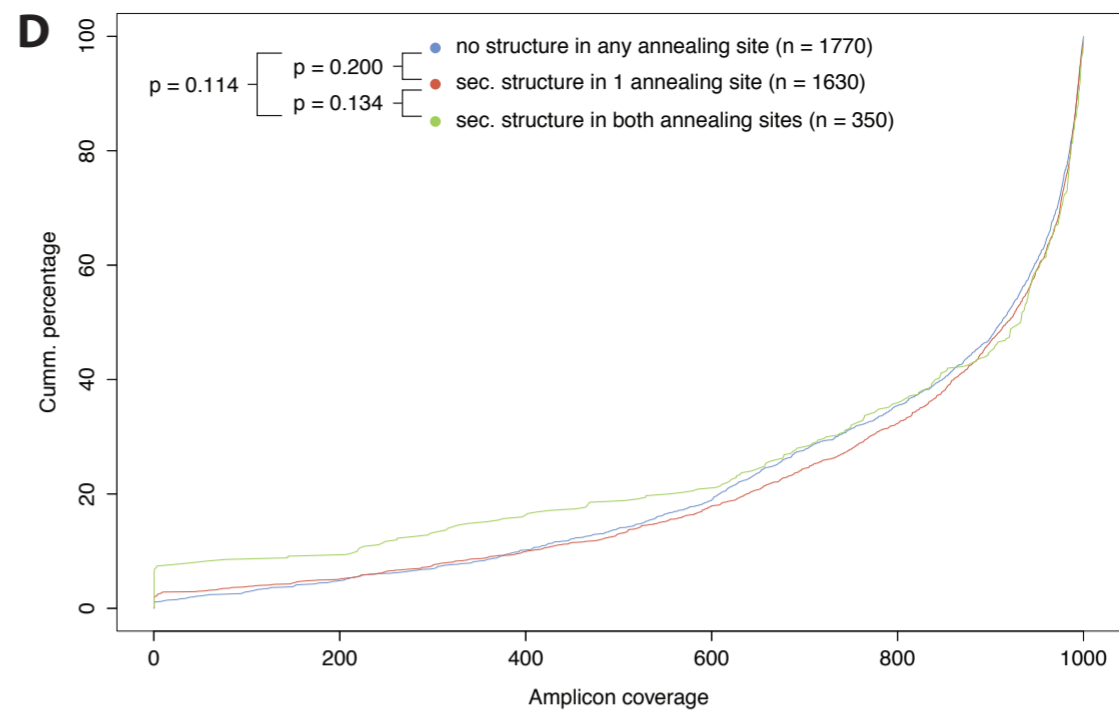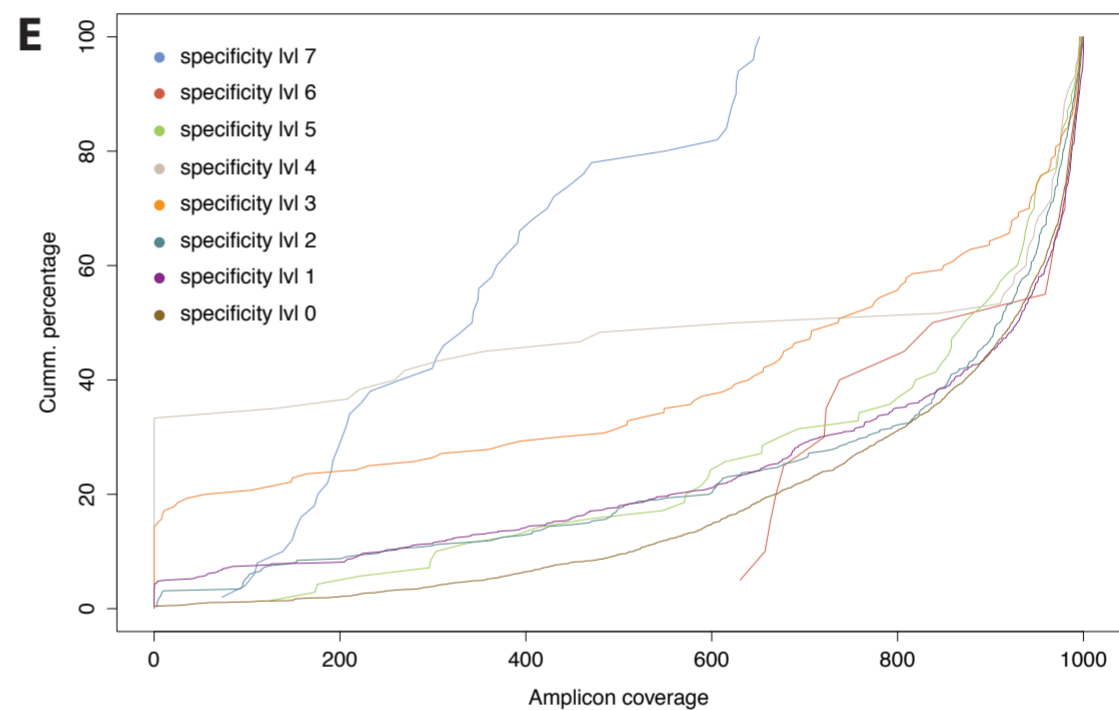

p-values for plot E :

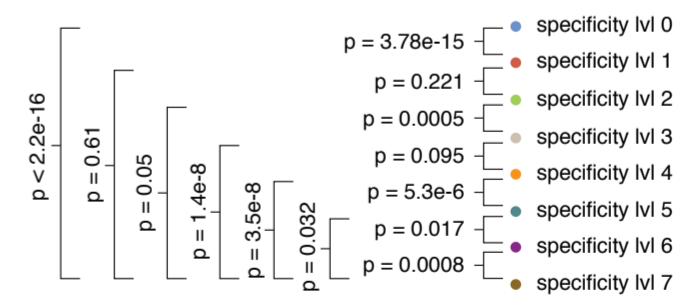

Supplement: Supplementary file 2 — Impact of assay features on sequencing coverage in project 3. Scatter plots of the assay sequencing coverage in function of A) the Gibbs free energy, B) the amplicon length and C) the amplicon GC content. Cumulative percentage plots of the assay sequencing coverage in function of D) the secondary structure content in primer annealing sites and E) the assay specificity level. Pearson correlation values and p values were calculated using the R functions cor() and ks.test() (Kolmogorov-Smirnov test) respectively. (PDF 1450 kb) [file 12859_2017_1809_MOESM2_ESM.pdf]

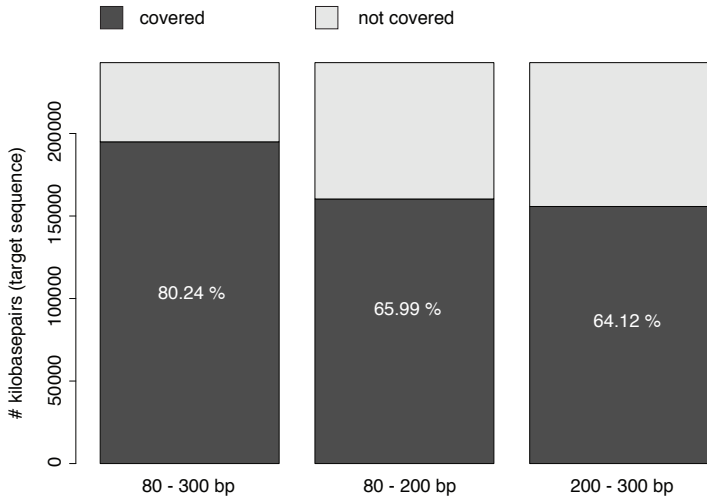

Supplement: Supplementary file 3 — Design performance when using amplicon sizes optimized for FFPE samples. Barplots showing target coverage percentages for 31 genes – totaling 242,939 nucleotides – using 80–200, 200–300 and 80–300 basepair design size ranges. (PDF 330 kb) [file 12859_2017_1809_MOESM3_ESM.pdf]
